# Identification and Mechanistic Analysis of a Novel Tick-Derived Inhibitor of Thrombin

**DOI:** 10.1371/journal.pone.0133991

**Published:** 2015-08-05

**Authors:** Willy Jablonka, Michalis Kotsyfakis, Daniella M. Mizurini, Robson Q. Monteiro, Jan Lukszo, Steven K. Drake, José M. C. Ribeiro, John F. Andersen

**Affiliations:** 1 Laboratory of Malaria and Vector Research, NIAID, National Institutes of Health, Rockville, Maryland, United States of America; 2 Institute of Parasitology, Academy of Sciences of the Czech Republic, České Budejovice, Czech Republic; 3 Instituto de Bioquimica Médica Leopoldo de Meis, Federal University of Rio de Janeiro, Rio de Janeiro, Brazil; 4 Research Technologies Branch, National Institute of Allergy and Infectious Diseases, National Institutes of Health, Rockville, Maryland, United States of America; 5 Critical Care Medicine Department, Clinical Center; National Institutes of Health, Bethesda, Maryland, United States of America; National Cerebral and Cardiovascular Center, JAPAN

## Abstract

A group of peptides from the salivary gland of the tick *Hyalomma marginatum rufipes*, a vector of Crimean Congo hemorrhagic fever show weak similarity to the madanins, a group of thrombin-inhibitory peptides from a second tick species, *Haemaphysalis longicornis*. We have evaluated the anti-serine protease activity of one of these *H*. *marginatum* peptides that has been given the name hyalomin-1. Hyalomin-1 was found to be a selective inhibitor of thrombin, blocking coagulation of plasma and inhibiting S2238 hydrolysis in a competitive manner with an inhibition constant (Ki) of 12 nM at an ionic strength of 150 mM. It also blocks the thrombin-mediated activation of coagulation factor XI, thrombin-mediated platelet aggregation, and the activation of coagulation factor V by thrombin. Hyalomin-1 is cleaved at a canonical thrombin cleavage site but the cleaved products do not inhibit coagulation. However, the C-terminal cleavage product showed non-competitive inhibition of S2238 hydrolysis. A peptide combining the N-terminal parts of the molecule with the cleavage region did not interact strongly with thrombin, but a 24-residue fragment containing the cleavage region and the C-terminal fragment inhibited the enzyme in a competitive manner and also inhibited coagulation of plasma. These results suggest that the peptide acts by binding to the active site as well as exosite I or the autolysis loop of thrombin. Injection of 2.5 mg/kg of hyalomin-1 increased arterial occlusion time in a mouse model of thrombosis, suggesting this peptide could be a candidate for clinical use as an antithrombotic.

## Introduction

Naturally occurring peptide and protein inhibitors of thrombin bind the enzyme at both the catalytic site and at surface regions known as exosites [1–5]. The active site is characterized by its catalytic triad consisting of His_57_, Asp_102_, Ser_195_ lying at the bottom of a deep cleft. The cleft is formed in part by the hydrophobic 60s-loop and the autolysis loop (centered approximately at residue 149) that act to limit access by potential substrates, creating a highly specific protease [1]. Two major positively-charged exosites are present, the fibrinogen-binding exosite (anion-binding exosite I) and the heparin binding exosite (anion-binding exosite II) that lie outside of the active site cleft on opposite sides of the molecular surface. Most substrates, including fibrinogen and PAR-1, bind at exosite I while exosite II is a binding site for heparin, platelets and the cofactor molecules FV and FVIII [4].

Thrombin inhibitors from blood-feeding animals bind in a variety of modes combining contacts at the active site and the anion-binding exosites. For example hirudin, an inhibitor from the medicinal leech *Hirudo medicinalis*, binds at the active site in a noncanonical (nonsubstrate-like) manner while the C-terminal portion of its peptide chain interacts with exosite I [6]. The exosite-binding region of hirudin has been combined with a substrate-like cleavage region to form hirulog, a potential therapeutic anticoagulant [7]. Ornithodorin from the tick *Ornithodoros moubata* contains two Kunitz-type domains, one of which binds in a hirudin-like, noncanonical way to the active site of thrombin while the other interacts with exosite I [8]. Haemadin, from the leech *Haemadipsa sylvestris*, binds the active site in a manner similar to hirudin, but its C-terminal portion is oriented differently and interacts with exosite II [9]. Triabin, a lipocalin-type inhibitor from the blood-feeding insect *Triatoma pallidipennis*, binds only at exosite I and does not inhibit the amidolytic activity of the enzyme on small-molecule substrates [10]. Variegin, from the saliva of the tick *Amblyomma variegatum*, is a relatively small 32-residue thrombin inhibitor that binds in a canonical (substrate-like) manner at the active site and is actually cleaved by the enzyme near its N-terminal end [11–13]. The C-terminal portion of the variegin chain exits the active site, binds at the “prime” subsites and continues along the thrombin surface to exosite I. The full-length peptide acts as a high-affinity, competitive inhibitor of thrombin while the C-terminal cleavage product acts as a noncompetitive inhibitor displaying lower binding affinity for the enzyme.

A second class of small, tick-derived thrombin inhibitors has been described from *Haemaphysalis longicornis* [14,15]. These peptides, known as madanins 1 and 2, were shown to inhibit coagulation and thrombin-mediated cleavage of macromolecular substrates, but did not inhibit hydrolysis of chromogenic substrates, and were suggested to interact only at an exosite [15]. In a subsequent study, madanins were found to inhibit chromogenic substrate cleavage at subphysiological salt concentrations, and to be cleaved by thrombin and FXa at multiple sites, suggesting interaction with the active site [14]. Unlike variegin, the cleavage products did not inhibit thrombin, and provided no information on possible exosite interactions. A crystal structure of the thrombin-madanin-1 complex, revealed a four-residue segment of madanin-1 bound in a canonical mode. The rest of the peptide was not visible due to disorder or was dissociated after cleavage [14].

In a previous study, the salivary gland transcriptome of the tick *Hyalomma marginatum rufipes* was characterized, and four transcripts, given the name hyalomins, were identified as having weak similarity to the madanins [16]. While the overall identity of the group in comparison with the madanins is low, the tripeptide sequence Pro-Arg-Leu near the C-terminus is conserved. The Arg-Leu peptide bond is a thrombin cleavage site in the madanins and the arginine residue occupies the P1 position of the peptide observed in the published crystal structure of the complex [14]. Here, we identify hyalomin-1, a 59-residue peptide having no cysteine residues, as an inhibitor of thrombin, and show that its mechanism of inhibition involves both active site and exosite interactions. We show that thrombin cleaves the peptide only at the conserved Arg-Leu peptide bond and that the C-terminal product is a noncompetitive inhibitor of chromogenic substrate cleavage. Additionally we demonstrate that a 24-residue fragment containing the cleavage site region and the C-terminal region inhibits thrombin in a competitive manner similar to the full-length peptide.

## Materials and Methods

### Materials

α-Thrombin was purchased from Sigma, Haematologic Technologies or purified after activation of prothrombin (Enzyme Research Laboratories) using *Oxyuranus scutellatus* venom. α-Chymotrypsin, plasmin and chymase were purchased from Sigma; β-tryptase was purchased from Promega, FXa was purchased from EMD Biosciences, FV, FX, FXI, FXIIa, γ-thrombin was purchased from Haematologic Technologies and from Enzyme Research Labs, kallikrein was purchased from Fitzgerald Industries International, elastase was purchased from Elastin Products, cathepsin G, FXIa, uPA, and tPA were purchased from Molecular Innovations, matriptase was from R&D Systems, proteinase 3 was from Merck and sequencing-grade trypsin was purchased from Roche. PT and APTT reagents were purchased from Stago Inc. Fibrinogen was purchased from Sigma-Aldrich. Polyphosphate, High MW (P700), was purchased from KeraFast.

### Peptide synthesis

Hyalomin-1, was synthesized by a stepwise, solid-phase method using Fmoc chemistry on an automated peptide synthesizer (Model 433A, Applied Biosystems, Life Technologies Corporation, Carlsbad, CA, USA). The N-terminal biotinylated derivative of this peptide was afforded by reacting a fully protected peptide-resin having a free N-terminal, with 2 equivalents of EZ-Link-NHS-LC-Biotin reagent (Pierce) and 4 equivalents of diisopropylethylamine (DIEA; Applied Biosystems) in dimethylformamide (DMF) for 3 hrs. After the trifluoroacetic acid cleavage step, synthetic peptides were purified to homogeneity by reversed-phase (RP) high-performance liquid chromatography (HPLC). The masses of both peptides were confirmed by MALDI mass spectrometry (AXIMA CFR+, Shimadzu Scientific Instruments, Inc., Columbia, MD, USA). The 01–41 peptide was synthesized by Atlantic Peptides (Lewisburg, PA) in both the biotinylated and nonbiotinylated forms. The 42–59 peptide was synthesized by Biosynthesis Co. (Lewisville, TX). The 36–59 peptide was synthesized by American Peptide Co. (Sunnyvale, CA), and the 13–44 peptide was synthesized by Atlantic Peptides.

### Screening for inhibition of serine proteases

Synthetic hyalomin-1 (1μM) was pre-incubated with a panel of serine proteases at 30°C for 10 min before addition of the corresponding substrate. Linear fits of fluorescence increases as a function of time were verified with the Magellan—Data Analysis Software (*Tecan* group Ltd), and the slope in arbitrary fluorescence units per second (r^2^ > 0.95) was used to calculate hydrolysis rate. Each experiment was performed in triplicate and the mean and standard error of three independent experiments were calculated. The substrate hydrolysis rate in the absence of the peptide was considered as 100% and compared with the remaining enzymatic activity in the presence of the peptide.

### Inhibition of coagulation

The APTT and PT were measured in a coagulometer (STart4, Stago Inc) using reconstituted lyophilized plasma (STA Coag- Control-N) with various peptides included at different concentrations. For the APTT 50 μl of platelet substitute and silica (STA PTT-A-5 reagent) were added and mixtures were pre-warmed. Reactions were started with the addition of 50 μl of 0.025M CaCl_2_. The PT was measured by pre-warming plasma with water or different concentrations of peptides and starting the reactions with 100 μl of calcium thromboplastin (STA Neoplastine-C1-Plus- 5 solution). Each measurement was done in triplicate.

### Inhibition of fibrin formation

Different concentrations of hyalomin-1 were incubated with 8 nM thrombin in 20 mM Tris pH 7.5, 150 mM NaCl buffer at 37°C for 10 min in 96 well flat bottom polystyrene plates. Fibrinogen was added to 2 mg/ml final concentration and absorbance was monitored at 650 nm at 10 second intervals for 30 minutes at 37°C in a microplate reader (Thermomax, Molecular Devices Inc.). Results of fibrin clotting were expressed as the time taken to reach an optical density of 0.01. Each experiment was repeated three times.

### Inhibition of platelet aggregation

Platelet-rich plasma (PRP) from healthy donors (NIH/CC/DTM) was centrifuged (1100 x *g*) for 15 minutes at 25°C after adding apyrase (0.4 U/ml). The platelet pellet was then resuspended in complete Tyrode buffer (5 mM HEPES pH 7.4, 137 mM NaCl, 2 mM KCl, 1mM MgCl_2_, 12 mM NaHCO_3_, 0.3 mM NaH_2_PO_4_, 5.5 mM glucose, 1.5 mg/ml BSA). Aggregation was monitored at 37°C in an aggregometer (Lumi-aggregometer, Chrono-log Corporation) by adding 100 μL of washed platelets to 200 μL of Tyrode buffer. Different concentrations of peptide were incubated at 37°C with washed platelets for 1 minute and aggregation was initiated by adding thrombin (3 nM). Each measurement was performed in duplicate or triplicate.

### Inhibition of thrombin-mediated activation of FXI

Inhibition of thrombin-mediated activation of FXI in the presence of long-chain (P700) polyphosphate (polyP) was done in according to Choi et al. [17] by adding 2 μM of polyP to buffer (20 mM Tris HCl, pH 7.4, 150 mM NaCl and 0.1% BSA) followed by different concentrations of peptide (23.4 nM to 600 nM). α-Thrombin (5 nM) and 60 nM of FXI were then added and samples were incubated at 37°C for 35 minutes. Reactions were stopped with 50 nM of the thrombin inhibitor anophelin [18,19] and polybrene to dissociate complexes with polyP. S2236 (Chromogenix Inc.) was added to a concentration of 250 μM and product formed was read as absorbance at 405 nM for 5–10 minutes in a microplate reader. Results were expressed as the percentage of the maximum activity (mAU/min) in the absence of peptide. Experiments were performed in duplicate.

### Inhibition of thrombin-mediated activation of FV

Human FV (4 μg) was incubated with thrombin (0.15 nM) in the presence or absence of 100 nM peptide at 37°C in 20 mM HEPES pH 7.4, 150 mM NaCl, 5 mM CaCl_2_, 0.1% PEG-6000 [20]. Reactions were stopped with gel loading buffer and the reaction products were visualized in an SDS-PAGE gel stained with Coomassie blue. The formation of FVa heavy chain was quantified by densitometry using ImageJ software (http://imagej.nih.gov/ij/, 1997–2014). The experiment was repeated three times.

### Inhibition of thrombin-mediated hydrolysis of S2338

The chromogenic substrate S2238 (Chromogenix Inc.) was incubated for 6 minutes at 37°C with different concentrations of hyalomin-1 or its derivatives in 20 mM Tris pH 7.4, 150 mM NaCl, 0.5% BSA pre-warmed at 37°C. Reactions were started by addition of thrombin (0.5 nM) and absorbance was monitored for 10 minutes at 405 nm in a microplate reader. The data were fit to competitive or non-competitive models of inhibition using GraphPad Prism 5. In both cases, nonlinear regression was used to fit to the hyperbolic equation:

v=Vmaxapp [S]/(Kmapp+[S])

In the case of competitive inhibition *Vmaxapp* = *Vmax* and in the case of noncompetitive inhibition *Kmapp* = *Km*.

In the case of competitive inhibition the inhibition constant (Ki) was obtained from fitting of: $Kmapp\;=\;Km\left(1+\frac{\left[I\right]}{Ki}\right)$.

For noncompetitive inhibition K_i_ was determined by fitting of: $Vmaxapp\;=\;Vmax/\left(1+\frac{\left[I\right]}{Ki}\right)$

The experiments were repeated three times.

### Surface plasmon resonance

Surface plasmon resonance (SPR) was used to measure interaction between immobilized peptides and thrombin using a Biacore T100 instrument. Approximately 500 resonance units of hyalomin-1 biotinylated at Lys_1_ or its N-terminal cleavage product (01–41) were bound to a neutravidin surface that had been immobilized on C5 chips using the amine coupling procedure. Kinetic measurements were carried out by injecting different concentrations of thrombin in 10 mM HEPES pH 7.4, 150 mM NaCl, 0.05%Tween-20. The chip surface was regenerated using injections of 10 mM glycine pH 2.5. Data were analyzed by global fitting of a 1:1 single state model using the Biacore T100 evaluation software. Binding of γ-thrombin with the same surface was also investigated under similar conditions.

### Mass spectral (MS) analysis of peptide cleavage

Hyalomin-1 (1–59), hyalomin-1 (36–59), hyalomin-1 (13–44) and hyalomin (13–44) sulfated were incubated at 25 μM without or with thrombin at 1 μM for 2 hours at 37°C in 1X TBS (10 X dilution of 10 X TBS pH 7.4, Molecular Biology Grade, Quality Biological, Inc). Reactions were stopped by freezing at –20°C until analysis. Samples (2 μl) were desalted in C-8 micro-columns (Ziptip-Milipore), washing 3 times with 0.1% TFA and eluting with 60% acetonitrile in water. Washes were diluted with 4 μl 50% acetonitrile and spotted (0.5–1 μl) on MALDI plates followed by addition of matrix solution. After drying, they were injected into the MS instrument and analyzed in reflectron (peptide plus thrombin) or linear (peptide alone) mode.

### Inhibition of carotid artery occlusion and bleeding in mice

All in vivo experiments described follow the rules for animal experimentation and care. Protocol IBqM081- 05/16 was approved by the Institute of Medical Biochemistry at Federal University of Rio de Janeiro (see S1 ARRIVE Checklist). Briefly, BALB/c mice were anesthetized (xylazine and ketamine) and the common carotid artery was isolated for measuring blood flow continuously using a 0.5-V Doppler flow probe coupled to a TS420 flow meter (Thransonic Systems, Ithaca, NY) [21]. Before induction of thrombosis the tail vein was injected with 50 μl of hyalomin-1 (1 and 2.5 mg/kg) or vehicle (PBS). Thrombi were formed by placing a piece of filter paper (1 x 2 mm) saturated with 7.5% FeCl_3_ on the adventitial surface or the artery for 3 minutes. After washing with normal saline, blood flow was continuously monitored for 60 minutes or until the blood flow dropped to zero for at least 10 seconds. Tail bleeding was performed on anesthetized mice injected intravenously with peptide (2.5 mg/kg) or vehicle alone (PBS) in 100 μl. After 15 minutes, the distal 2 mm segment was removed and the tail was immersed in 40 ml distilled 37°C warmed water. Absorbance at 540 was measured to estimate hemoglobin content.

### Statistical analysis

Paired *t*-tests were used for statistical analysis of the observed inhibition in serine protease screening in the presence of peptide. Statistical significance was considered when p < 0.05 comparing controls to peptide treated groups. ANOVA with Dunnett’s Multiple Comparison test was used for analysis of occlusion time and unpaired *t*-test was used for bleeding test analysis. Statistical significance was considered if p < 0.05 comparing controls to peptide treated groups.

## Results and Discussion

### Hyalomin-1, a specific inhibitor of thrombin

**Fig 1:**
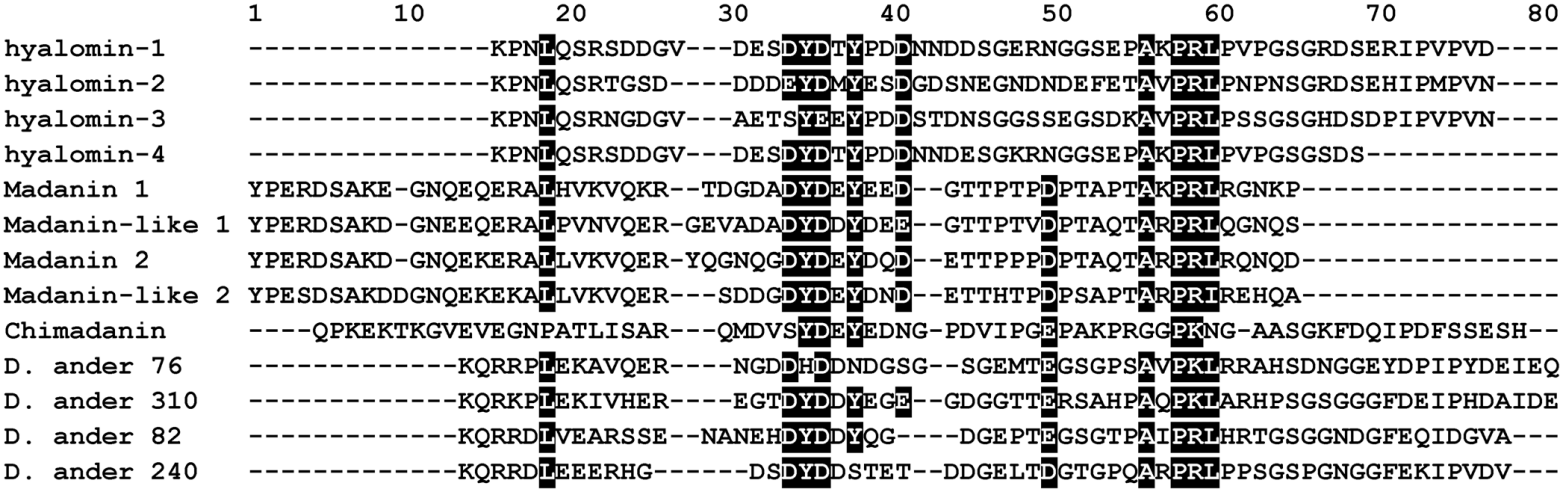
Alignment of hyalomins from *H*. *marginatum rufipes* with the madanins from *H*. *longicornis* and similar sequences from *D*. *andersoni*. Signal peptide sequences have been removed, and regions of conservation are highlighted in black. Accession numbers from the Genbank database are: GI:307006449, GI:307006483, GI:307006445 and GI:307006427 for hyalomins 1–4, GI:30025562 and GI:30025564 for madanins 1 and 2, GI:67906166 and GI:67968369 for madanin-like peptides 1 and 2. D. ander 76, 310, 82 and 240 sequences are found in Francischetti et al, 2009 support material.

The salivary gland transcriptome from the hard tick *Hyalomma marginatum rufipes* contains four sequences encoding peptides, designated as hyalomins-1-4, that show weak similarity to the thrombin inhibitors madanin 1 and 2 from *Haemaphysalis longicornis* (Fig 1) [16]. The peptides in this group (including four from a third tick species, *Dermacentor andersoni*) [22] contain similar numbers of amino acids, but differ in length at either their N- or C-termini, leaving an approximately 50-residue shared central core region having low overall identity (Fig 1). The madanins have an extended N-terminal region compared with peptides from the other genera while the hyalomins and the *D*. *andersoni* peptides are extended at the C-terminus (Fig 1) [15]. Chimadanin, a second form of thrombin inhibitor from *H*. *longicornis* is extended at both the N and C-termini (Fig 1) [23]. The central core of these peptides contains a weakly conserved acidic region that lies N-terminal to a Pro-Arg-Leu motif and forms a putative serine protease cleavage site (Fig 1).

**Fig 2:**
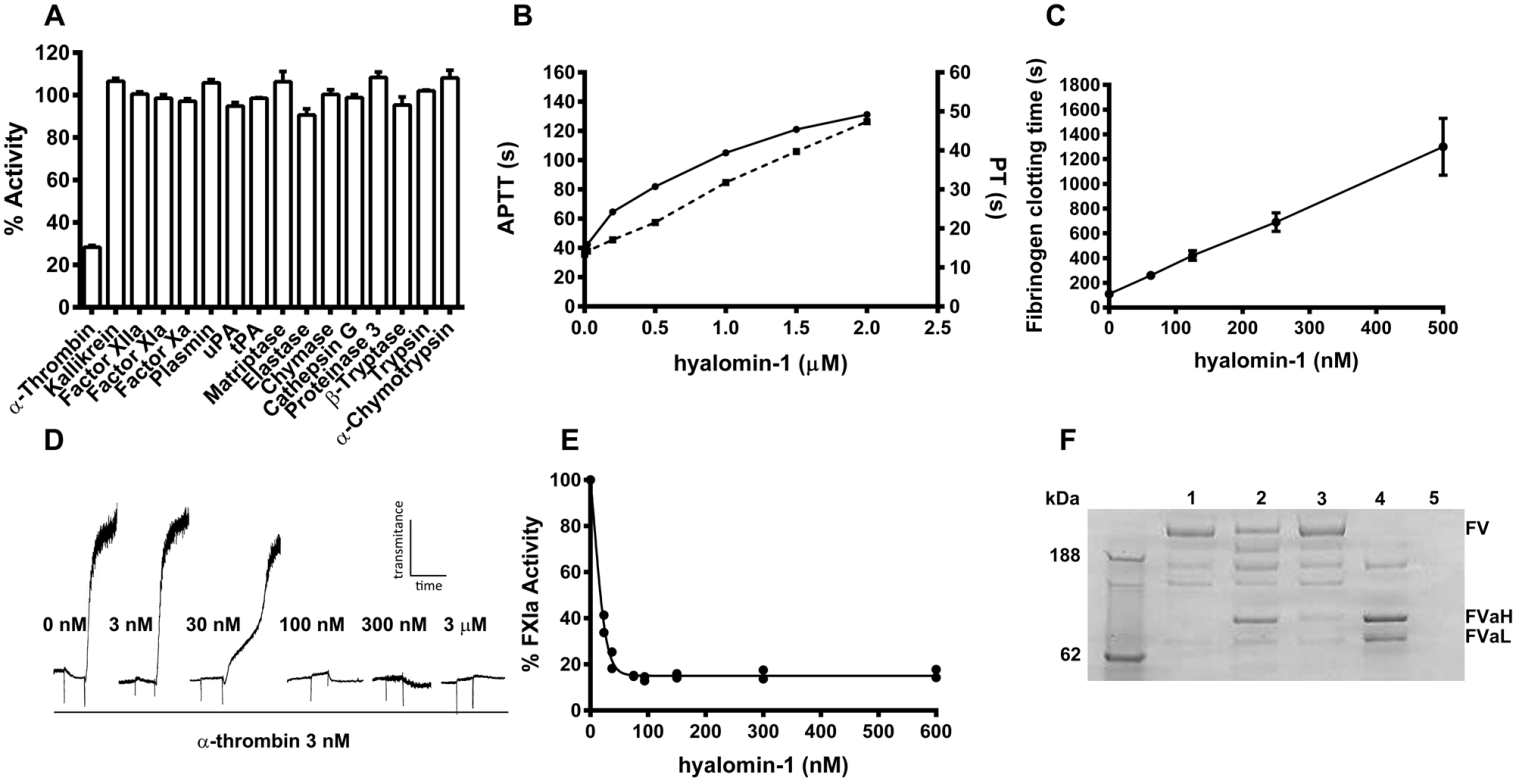
Hyalomin-1 is a specific thrombin inhibitor. (A) The activity of 16 serine proteases in the presence of hyalomin-1 (1 μM) relative to their activity in the absence of inhibitor. (B) Coagulation time of human plasma incubated with hyalomin-1 as measured using the APTT (solid line), and PT (dashed line) assay procedures. (C) Conversion of fibrinogen to fibrin by thrombin in the presence of increasing concentrations of hyalomin-1 as indicated by seconds for increase in absorbance to 0.01 at 650 nm. (D) Aggregation of washed platelets induced by thrombin in the presence of various concentrations of hyalomin-1, as measured by an increase in transmittance in an aggregometer. (E) Polyphosphate-activated cleavage of FXI by thrombin in presence of hyalomin-1. FXIa was measured by hydrolysis of the chromogenic substrate S2236. (F) FV cleavage by thrombin in the presence and absence of hyalomin-1 as measured by SDS-PAGE. Lane 1 –FV alone after 60 min incubation. Lane 2 –FV and thrombin after 10 minutes incubation. Lane 3 –FV, thrombin and hyalomin-1 after 10 minutes incubation. Lane 4 –FV and thrombin after 60 minutes incubation. Lane 5 –thrombin alone.

Hyalomin-1 was synthesized without its putative signal sequence and tested for inhibition of eight coagulation proteases along with eight additional important serine proteases having functions not related to coagulation (Fig 2A). In each case, the effect of the peptide on the rate of hydrolysis of an appropriate fluorogenic substrate was evaluated. Only thrombin (α-thrombin throughout manuscript unless otherwise indicated) was significantly inhibited in these tests, losing 72% of its control activity in the presence of the peptide (Fig 2A). In a manner consistent with these observations, the coagulation time of recalcified human plasma in the APTT and PT assays was prolonged by 3.4 and 3.5-fold, respectively, at a concentration of 2 μM hyalomin-1 when compared to untreated controls (Fig 2B). The thrombin-catalyzed formation of fibrin from purified fibrinogen was also inhibited by hyalomin-1. The time required to convert purified fibrinogen to a fibrin gel, as indicated by time in seconds to an increase in the optical density at 650 nm, increased 11.8-fold at a hyalomin-1 concentration of 500 nM compared to a peptide-free control (Fig 2C). Together, these data indicate that hyalomin-1 is a specific inhibitor of thrombin capable of significantly delaying the formation of a fibrin clot in whole plasma.

### Inhibition of cleavage of physiological targets of thrombin by hyalomin-1

In addition to the cleavage of fibrinogen, thrombin catalyzes a number of other important proteolytic reactions related to hemostasis. We tested the ability of hyalomin-1 to inhibit thrombin-mediated activation of additional macromolecular substrates involved in coagulation, platelet activation and fibrinolysis. The protease activated receptors PAR-1 and PAR-4 on platelets are cleaved by thrombin leading to activation and subsequent aggregation [24]. Inhibition of PAR cleavage by hyalomin-1 was demonstrated by measuring its effect on the aggregation of washed platelets initiated by thrombin. A detectable delay in aggregation was observed in the presence of 30 nM hyalomin-1 and 3 nM thrombin where maximal aggregation was seen only as a secondary wave response (Fig 2D). At peptide concentrations of 100 nM and above, aggregation was almost completely inhibited.

Thrombin also participates in a number of biochemical feedback reactions involving the activation of proteases and cofactor proteins, such as FXI and FV, which serve to amplify its own generation. In one of these reactions, polyP-stimulated thrombin efficiently converts FXI to its active form FXIa [17]. We examined the inhibitory effect of hyalomin-1 on this reaction by measuring FXIa activity after incubation in a reconstituted system containing thrombin, long-chain polyP and FXI. The peptide was found to reduce FXIa generation to a low level in a concentration-dependent manner with an IC_50_ value of approximately 25 nM (Fig 2E). As shown in Fig 2A, the peptide does not inhibit the amidolytic activity of FXIa itself, indicating that it is acting solely through the inhibition of thrombin.

FV is cleaved by thrombin to form FVa, an essential cofactor component of the prothrombinase complex. We tested the ability of hyalomin-1 to inhibit the activation of FV using SDS-PAGE analysis of cleavage products generated in a reconstituted system (Fig 2F). When FV was incubated with thrombin in the absence of hyalomin-1 for 10 minutes at 37°C, prominent bands appeared corresponding to the heavy and light chains of FVa along with intermediate products (Fig 2F). The conversion to FVa proceeds to a larger extent after incubation for 60 minutes (Fig 2F). Densitometric measurements revealed that in the absence of hyalomin-1, 55% of FV was converted to FVa in 10 minutes (Fig 2F). When hyalomin-1 was added to the system, the quantity of FV remained essentially unchanged but a small increase in the intensity of bands representing the FVa heavy and light chains was detectable (Fig 2F). These results indicate that hyalomin-1 effectively inhibits the conversion of FV to FVa by thrombin.

### Mechanistic analyses of hyalomin-1 inhibition

**Fig 3:**
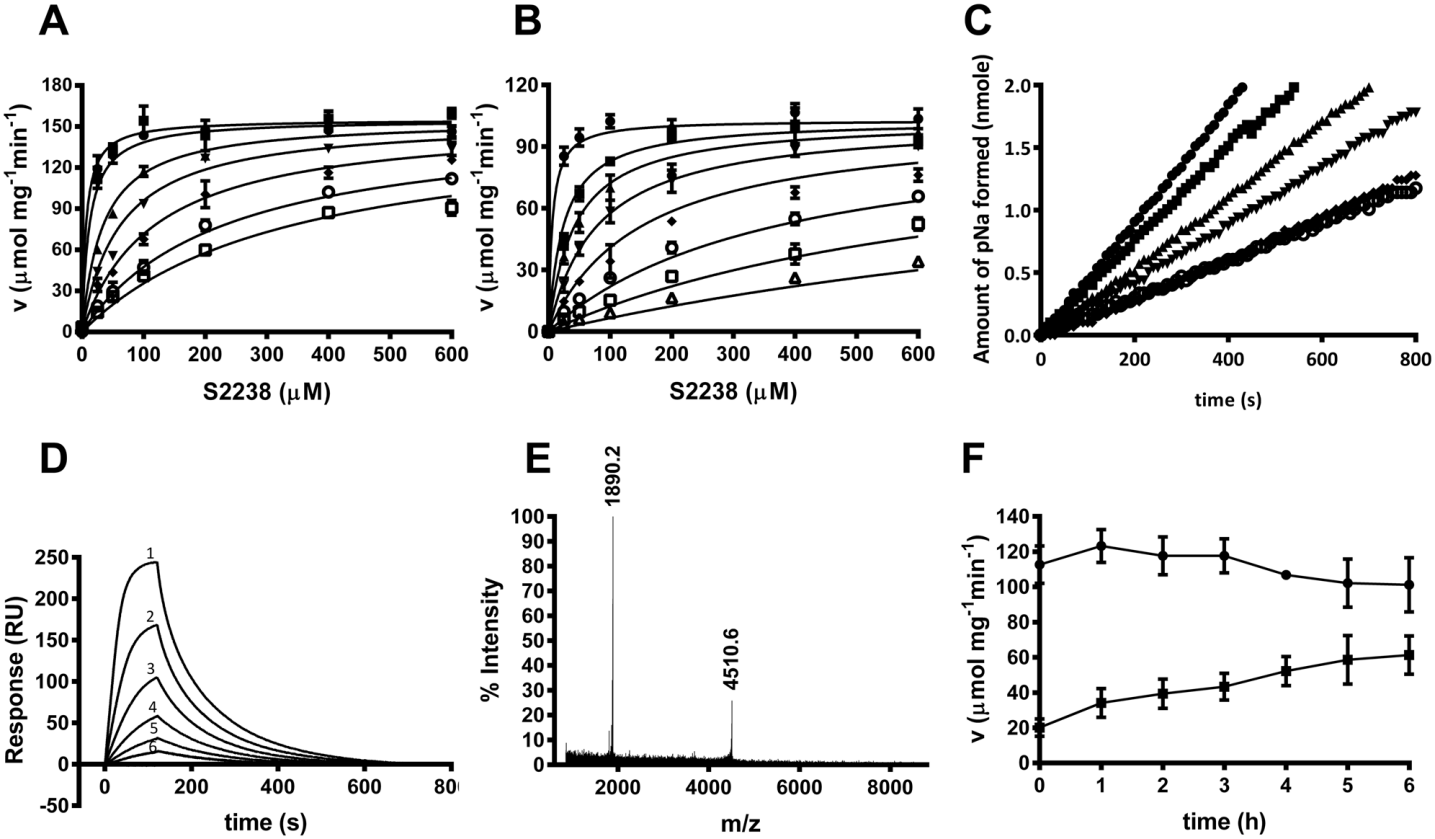
Hyalomin-1 is a competitive fast binding classical inhibitor of thrombin that is also cleaved by thrombin. (A) Kinetics of S2238 hydrolysis by thrombin in the presence of increasing concentrations of hyalomin-1 at a sodium chloride concentration of 150 mM. Inhibitor concentrations: 0 nM (filled circles), 10 nM (filled squares), 50 nM (filled triangles), 100 nM (filled inverted triangles), 200 nM (filled diamonds), 400 nM (open circles), 600 nM (open squares). (B) Experiment performed as in panel A but at a sodium chloride concentration of 50 mM. Hyalomin-1 concentrations: 0 nM (filled circles), 1.25 nM (filled squares), 2.5 nM (filled triangles), 5 nM (filled inverted triangles), 10 nM (filled diamonds), 25 nM (open circles), 50 nM (open squares), 100 nM (open triangles). (C) Progress curves of thrombin activity in the presence of hyalomin-1: 0 nM (filled circles), 50 nM (filled squares), 100 nM (filled triangles), 200 nM (filled inverted triangles), 400 nM (filled diamonds), 600 nM (open circles). (D) Measurement of thrombin binding to immobilized hyalomin-1 by SPR. Sensograms were obtained by injection of thrombin at concentrations of 50 nM (1), 25 nM (2), 12.5 nM (3), 6.25 nM (4), 3.125 nM (5) and 1.563 nM (6). Kinetic constants are indicated in the text. (E) Mass spectral analysis of hyalomin-1 cleavage products after incubation with thrombin for 2 h at 37°C. The mass values on the graph correspond to cleavage at the Arg_41_-Leu_42_ peptide bond (peptides 01–41 and 42–59 in Fig 5A). (F) Effect of incubation time at 37°C on the inhibition of thrombin (0.5 nM) by hyalomin-1 (400 nM) in the presence of 50 μM S2238. The activity of thrombin in the absence of hyalomin-1 is shown as filled circles, while activity in the presence of hyalomin-1 is shown as filled squares.

Kinetic analyses showed hyalomin-1 to be a competitive inhibitor of S2238 hydrolysis by thrombin (Fig 3A and 3B) with inhibition constants (K_i_) of 11.9 ± 1.8 nM and 0.4 ± 0.1 nM at salt concentrations of 150 and 50 mM, respectively, as compared to reported values of 56 nM for madanin-1 and 32 nM for madanin-2 at an ionic strength of 50 mM (14). The linear progress curves obtained immediately after thrombin addition indicated rapid equilibrium binding of hyalomin-1, making it a “fast binding” inhibitor (Fig 3C). Binding to thrombin was also evaluated by surface plasmon resonance (SPR) using hyalomin-1 immobilized through a biotin moiety added to the N-terminal lysine residue of the peptide (Fig 3D). Analysis of the concentration-dependent kinetics of thrombin binding to this surface produced a second order association rate constant (k_a_) for thrombin binding of 1 x 10^6^ M^-1^s^-1^ and a dissociation rate constant (k_d_) of 0.02 s^-1^. The calculated dissociation equilibrium constant (K_D_) of 19 nM was consistent with the K_i_ obtained from steady-state kinetic results.

The Pro-Arg_41_-Leu motif is the most highly conserved region in the amino acid alignment from the three tick species shown in Fig 1. Madanins-1 and 2 are known to be cleaved by thrombin at the Arg_54_-Leu_55_ peptide bond contained within this motif [14]. Interestingly, they were also cleaved upstream of this site at Lys_21_, a residue that is not conserved in the hyalomins. Additionally, the madanins are cleaved by factor Xa at Arg_16_, Arg_25_ and Arg_54_. The former two positions are not conserved in the hyalomins, while the latter is equivalent to Arg_41_, the thrombin cleavage site [14]. When hyalomin-1 was incubated with thrombin for two hours at 37°C two fragments were observed by mass spectrometry having masses corresponding to residues 1–41 (m/z 4510.6) and 42–59 (m/z 1890.2), indicating that the Arg_41_-Leu_42_ peptide bond is the sole cleavage site. No full-length peptide (m/z 6381.3) remained at the end of the incubation period, demonstrating that cleavage proceeded to completion at a 25:1 peptide to enzyme ratio (Fig 3E). When the activity of thrombin was monitored at a 800:1 peptide to enzyme ratio, the potency of the inhibitor diminished in a time-dependent manner over a six hour incubation period, consistent with a loss of activity due to cleavage (Fig 3F).

**Fig 4:**
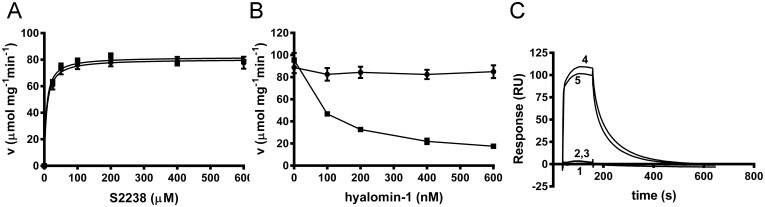
Hyalomin-1 does not inhibit or interact with γ-thrombin. (A) Steady state kinetic analysis of γ-thrombin in the absence (filled circles) or presence (filled squares) of 600 nM hyalomin-1. (B) γ-Thrombin (black circles) and α-thrombin (black squares)-catalyzed hydrolysis S2238 (50 μM) in the presence of hyalomin-1. (C) Binding of γ-thrombin and α-thrombin to immobilized hyalomin-1 measured by SPR. Buffer alone (1), 50 nM γ-thrombin (2), 100 nM γ-thrombin (3), 50 nM α-thrombin (4), 100 nM α-thrombin (5).

As is the case with other peptide inhibitors of thrombin, the binding of hyalomin-1 is most likely stabilized by exosite interactions. γ-Thrombin is generated through cleavages of the B chain of α-thrombin at Arg_75_ and Lys_149E_ that disrupt the structures of the autolysis loop in the vicinity of the catalytic site, and exosite I, the fibrinogen binding site. These changes produce an enzyme form that hydrolyzes S2238 normally, but is incapable of cleaving fibrinogen due to the loss of exosite surfaces [25]. We determined kinetic parameters for γ-thrombin cleavage of S2238 and found them to be essentially identical to those seen with α-thrombin (Fig 4A). However, hyalomin-1 did not inhibit hydrolysis of the chromogenic substrate by γ-thrombin at peptide concentrations of up to 600 nM (Fig 4A), while α-thrombin was strongly inhibited under this same concentration and conditions (Fig 4B). γ-Thrombin also showed no detectable binding to immobilized hyalomin-1 in SPR experiments, while α-thrombin exhibited high levels of binding to the same surface (Fig 4C). These results suggest that disruption of the thrombin structure in the vicinity of the autolysis loop and exosite I abrogated hyalomin-1 binding, thereby implicating these areas as potential binding sites for the peptide.

**Fig 5:**
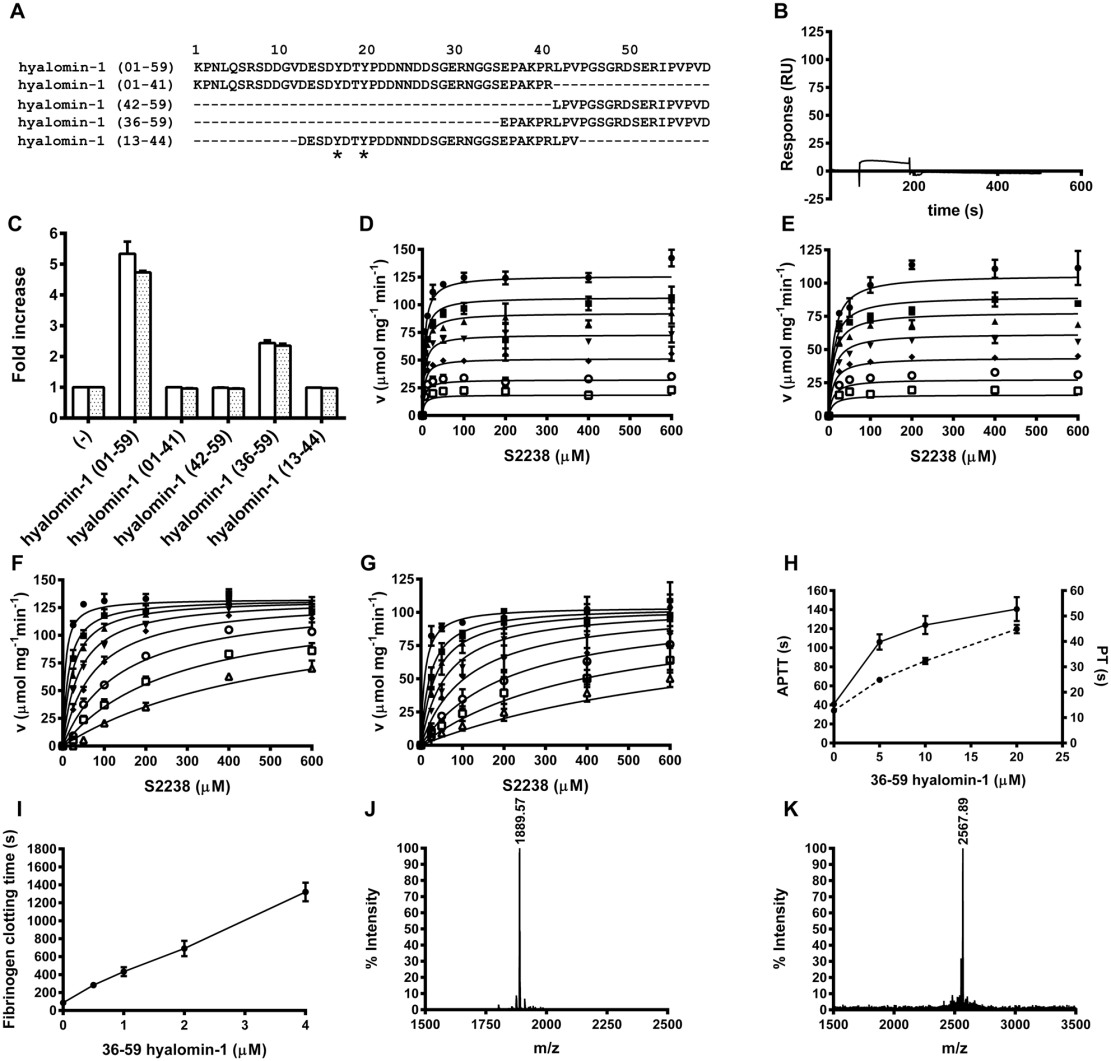
Activity of hyalomin-1 cleavage products and truncated forms. (A) Alignment of full length hyalomin-1 (01–59) with its thrombin cleavage products (01–41 and 42–59) as well as two other truncated peptides (36–59 and 13–44) that include the Pro-Arg-Leu thrombin cleavage site. Asterisks denote tyrosine sulfation of the sulfated form of the 13–44 peptide (B) SPR analysis of thrombin binding with immobilized 01–41 peptide. (C) Inhibition of coagulation of plasma by hyalomin-1 and derivative peptides. Clotting time in the PT (white bars) and APTT (hatched bars) assays are normalized to a value of 1 in the absence of peptide. (D) Steady state kinetic analysis of inhibition of thrombin-catalyzed hydrolysis of S2238 by the 42–59 peptide in buffer containing 150 mM NaCl. Inhibitor concentrations: 0 μM (filled circles), 0.313 μM (filled squares), 0.625 μM (filled triangles), 1.25 μM (filled inverted triangles), 2.5 μM (filled diamonds), 5 μM (open circles), 10 μM (open squares) (E) Experiment in panel D performed in buffer containing 50 mM NaCl. (F) Kinetics of thrombin-catalyzed hydrolysis of S2238 in the presence of hyalomin-1 (36–59) in buffer containing 150 mM NaCl. Inhibitor concentrations: 0 μM (filled circles), 0.13 μM (filled squares), 0.26 μM (filled triangles), 0.51 μM (filled inverted triangles), 1 μM (filled diamonds), 2 μM (open circles), 4.1 μM (open squares), 8.2 μM (open triangles) (G) The experiment of panel F performed in buffer containing 50 mM NaCl. (H) Coagulation time of human plasma incubated with hyalomin-1 (36–59) as measured using the APTT (solid line) and PT (dashed line) assay procedures. (I) Fibrinogen clotting time by thrombin at increasing concentrations of hyalomin-1 (36–59) as indicated by time (s) taken to reach an absorbance value of 0.01 at 650 nm. (J) and (K) Mass spectral analysis of hyalomin-1 (36–59) incubated with thrombin (J) or alone (K).

In order to further understand the structural determinants of hyalomin-1 binding, we synthesized the two peptide cleavage products and tested their activity in enzymatic and binding assays (Fig 5A). The 01–41 fragment, containing the putative P1 residue Arg_41_, did not inhibit coagulation of plasma (Fig 5C) or hydrolysis of S2238 (Data not shown) at concentrations of up to 5 μM. SPR analysis of thrombin binding with immobilized, biotinylated 01–41 peptide also revealed no detectable interaction of 200 nM thrombin with this surface (Fig 5B). The 42–59 peptide was also inactive in coagulation assays at concentrations up to 5 μM (Fig 5C), but was found to inhibit S2238 hydrolysis when incubated with thrombin at concentrations above 300 nM (Fig 5D). Kinetic analysis showed the 42–59 peptide to be a non-competitive inhibitor of S2238 hydrolysis with a calculated Ki value of 1.7 ± 0.1 μM at an ionic strength of 150 mM (Fig 5D). The kinetic parameters did not change significantly (Ki = 1.8 ± 0.1 μM) when the salt concentration was reduced to 50 mM (Fig 5E). This peptide also had no effect on hydrolysis of S2238 by γ-thrombin (Data not shown), suggesting that the C-terminal region of hyalomin-1 interacts with thrombin in the vicinity of the autolysis loop, or possibly at exosite I. However, the relatively short length of the C-terminal fragment along with its lack of negatively charged residues may make it less likely to extend as far as exosite I and interact with its positively-charged surface.

The properties of a hyalomin-1 derivative containing the region surrounding the scissile peptide bond (Arg_41_-Leu_42_) as well the C-terminal portion of the mature peptide were also evaluated (Fig 5A). This variant (36–59) contained the P1 residue Arg_41_, the P2-P6 residues, and the entire sequence of the 42–59 fragment. The 24-residue peptide was found to inhibit coagulation of recalcified plasma, cleavage of fibrinogen, and hydrolysis of S2238 (Fig 5C, 5F–5I). Kinetic analysis of S2238 cleavage showed the 36–59 peptide to be a competitive inhibitor, exhibiting a Ki value of 100 ± 2 nM at an ionic strength of 150 mM, suggesting that it binds to thrombin with approximately ten-fold lower affinity than hyalomin-1 but inhibits by a similar mechanism (Fig 5F). Unlike the full-length hyalomin-1, the kinetic parameters for cleavage of S2238 did not change significantly at a salt concentration of 50 mM (Ki = 0.18 ± 0.03 μM) indicating that sequences upstream of the cleavage site in hyalomin-1, particularly the acidic region, may also play a role in the salt concentration-dependent binding of the full-length form (Fig 5G). The coagulation time of recalcified plasma in the APTT and PT assays were also prolonged 3.4 and 3.5-fold (Fig 5H), respectively, at a concentration of 20 μM, indicating a 10-fold lower activity than full-length hyalomin-1 (compare to Fig 2B). Also the 36–59 peptide inhibited the cleavage of fibrinogen by thrombin (Fig 5I), but required about 6-fold higher concentration than hyalomin-1 to produce a comparable effect (compare to Fig 2C). After incubation with thrombin for two hours at 37°C, mass spectral analysis showed complete digestion of the 36–59 peptide, with the appearance of a fragment at m/z 1889.6 indicative of the 42–59 peptide as a product (Fig 5J) while a thrombin-free control showed no cleavage (Fig 5K). This result verified that like the full-length inhibitor, the Arg_41_-Leu_42_ peptide bond is the only site of cleavage.

**Fig 6:**
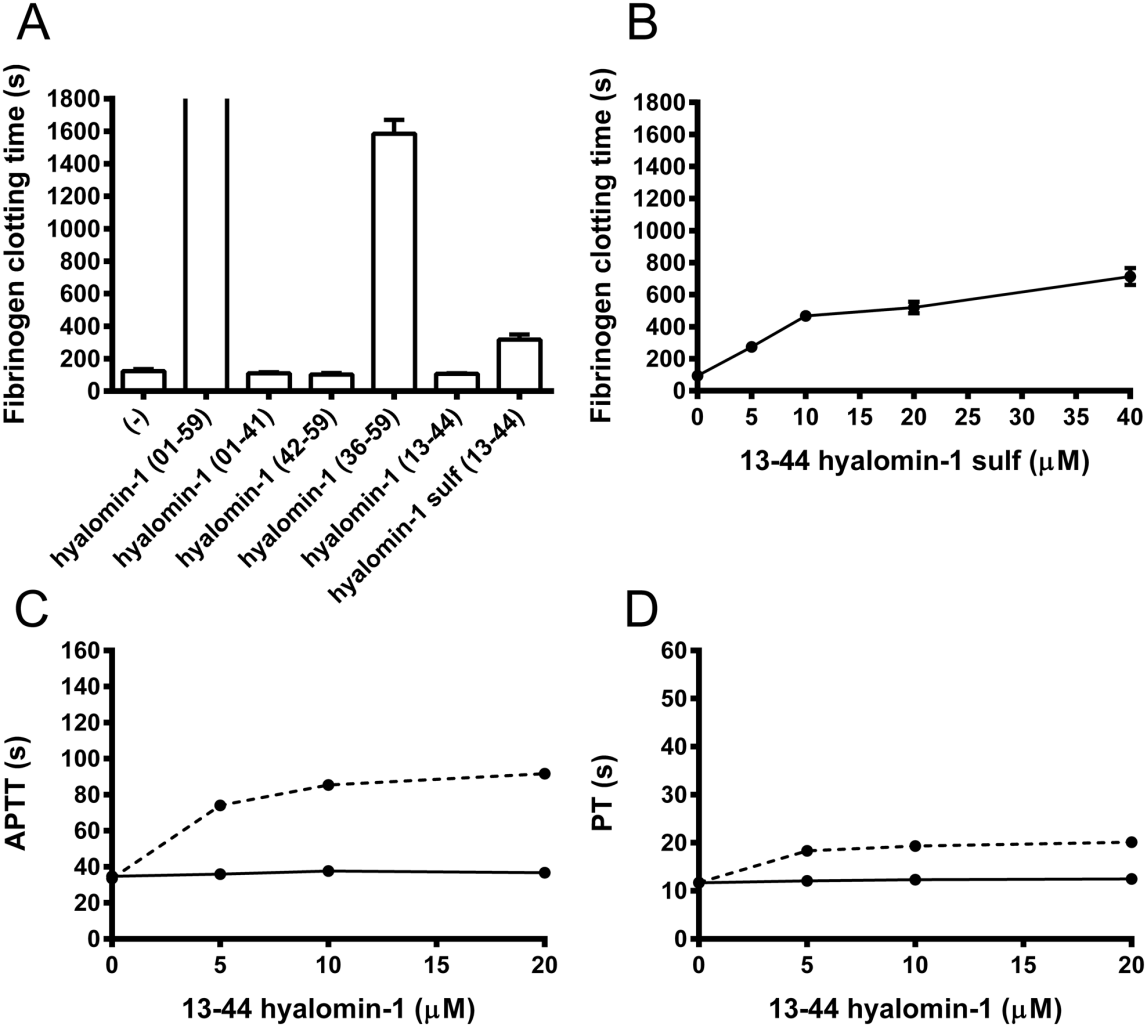
Sulfation of hyalomin-1 (13–44) truncated form results in a modest increase in potency. (A) Conversion of fibrinogen to fibrin by thrombin in the presence of different hyalomin-1 truncated forms (5 μM) as indicated by time in seconds for increase in absorbance of 0.01 at 650 nm. Bars represent mean with SE. Full hyalomin-1 (01–59) completely inhibited fibrinogen clotting during the time assayed. (B) Same assay as in A but at different concentrations of hyalomin-1 (13–44) sulfated. (C) APTT and (D) PT assay procedures for coagulation time of human plasma in the presence of different concentrations of hyalomin -1 (13–44) sulfated (dashed lines), or hyalomin-1 (13–44) solid lines.

Finally, we tested a 13–44 peptide variant that contains the conserved acidic region N-terminal to the cleavage site as well as the cleavage region including the P1´-P3´ residues (Fig 5A). This peptide did not inhibit coagulation of plasma (Fig 5C) or cleavage of S2238 at concentrations up to 5 μM (data not shown), but was hydrolyzed by thrombin at the predicted site after 2 hr at a peptide concentration of 25 μM and a thrombin concentration of 1 μM (data not shown). This indicates that the region upstream of the cleavage site interacts only weakly with thrombin, but does interact with the active site. Two potential tyrosine sulfation sites [26] are present in this peptide at positions 17 and 20 (in 1–59 numbering) (Fig 5A), leading us to synthesize a variant of the 13–44 peptide with sulfotyrosine replacing tyrosine at these positions. The sulfated peptide only weakly inhibited the cleavage of fibrinogen and coagulation of plasma (Fig 6), but did not inhibit hydrolysis of S2238 even at elevated concentrations (data not shown). However, the peptide was hydrolyzed by thrombin at the predicted cleavage site (data not shown), indicating that it must interact weakly at the thrombin active site, The reduction in potency of hyalomin-1 at high salt concentrations and the lack of salt sensitivity of inhibition by the 36–59 variant suggests that ionic interactions of the highly charged region upstream of the cleavage site may enhance affinity of the full length peptide at lower salt concentrations, but the region C-terminal to the cleavage site remains essential to this interaction, and little inhibition occurs in its absence. Any sulfation of tyrosine occurring in the salivary gland may assist the interaction of the peptide with the enzyme, but the effect of this modification does not appear to be large.

### Hyalomin-1 inhibits thrombosis *in vivo*

**Fig 7:**
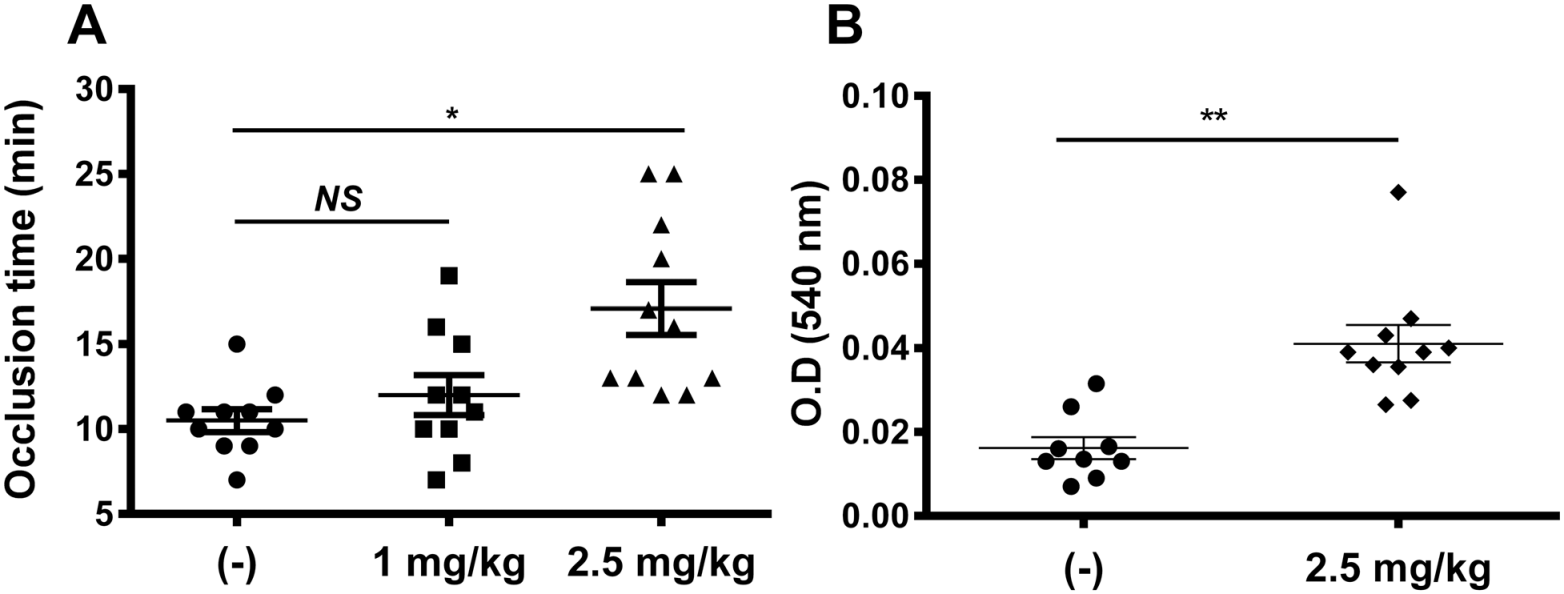
Hyalomin-1 exhibits antithrombotic activity in vivo. (A) Occlusion of the carotid artery in BALB/c mice induced by FeCl_3_ and measured by laser Doppler flowmetry after injection of vehicle alone or hyalomin-1 at doses of 1 and 2.5 mg/kg. (B) Bleeding from the tail of BALB/c mice after injection of vehicle alone or vehicle plus hyalomin-1 at a dose of 2.5mg/kg. Statistical significance levels: * P < 0.05, ** P < 0.001.

In order to assess the potential of hyalomin-1 as an antithrombotic agent, *in vivo* thrombosis assays were carried out in mice. Injury of the carotid artery was induced by ferric chloride after intravenous injection of hyalomin-1 at concentrations of 1 or 2.5 mg/kg and the time to arterial occlusion was measured. Hyalomin-1 produced a detectable delay in occlusion at 1 mg/kg and a statistically significant 1.6 fold delay at 2.5 mg/kg peptide (Fig 7A).

Next, the effect of hyalomin-1 on bleeding was estimated using the tail transection method. Fig 7B shows that injection of hyalomin-1 (2.5 mg/kg) produces significant bleeding as would be expected for an inhibitor of thrombin.

## Conclusions

Although it shows only weak similarity to the madanins, hyalomin-1 inhibits thrombin by a similar mechanism, and is cleaved by the enzyme at the homologous Arg-Leu peptide bond contained within the Pro-Arg-Leu motif near the C-terminus of the peptide. In contrast to the madanins whose cleavage products are not inhibitory, the C-terminal cleavage product of hyalomin-1 inhibits the amidolytic activity of thrombin in a noncompetitive manner suggesting that this fragment binds at an enzyme exosite. Furthermore, a peptide variant containing only residues in the vicinity of the cleavage site and the C-terminal region has similar inhibitory properties to the full length peptide, and is cleaved by thrombin, but shows an approximately 10-fold reduction in potency. The inhibitory mechanism of hyalomin-1 appears similar to that of variegin, a thrombin inhibitor from the tick *Amblyomma variegatum*, although the amino acid sequences of the two peptides show no apparent amino acid similarity. The 32-residue variegin sequence has a Pro-Lys-Met motif near the N-terminal end of the peptide and is cleaved at the Lys_10_-Met_11_ peptide bond. Like hyalomin-1, the C-terminal cleavage product of variegin inhibits thrombin with reduced potency relative to the full-length peptide and shows a noncompetitive inhibitory mechanism [11,12]. Variegin binds thrombin with higher affinity than hyalomin-1, however, making it a more potent inhibitor. In the crystal structure of the variegin-thrombin complex the C-terminal cleavage product is bound at the prime sites and exosite I of thrombin, and conformational changes in the catalytic triad residues were postulated to be responsible for the observed noncompetitive inhibition [13].

A previously described crystal structure of the madanin-1-thrombin complex shows a four-residue madanin peptide sequence Ala_51_-Lys_52_-Pro_53_-Arg_54_ bound in a substrate-like (canonical) manner at the catalytic site with Arg_54_ occupying the P1 position [14]. This binding mode indicates that the C-terminal cleavage product would be oriented toward exosite-1 in the full-length peptide but has been lost in the crystal, probably due to cleavage. The lack of inhibition of γ-thrombin by hyalomin-1 or its derivatives, along with the inhibitory properties of its fragments, suggests that the C-terminal part of hyalomin-1 interacts in the region of exosite-1 or the autolysis loop in addition to the catalytic site. These results are consistent with a hyalomin-1 binding mode for the region surrounding the P1 residue that is similar to that seen in the madanin-thrombin crystal structure. Remarkably, the amino acid sequence on the C-terminal side of the scissile bond is not well conserved among the peptides shown in Fig 1 suggesting that the region may not play the same role in other related sequences. Having only six amino acid residues on the C-terminal side of the cleavage site, the madanins do not appear to have the length necessary to bridge the distance between the catalytic site and exosite I, while hyalomin-1, with 18 C-terminal residues could potentially span this distance. However, the C-terminal end of hyalomin-1 contains fewer negatively charged residues than seen in variegin, hirulog or other exosite I-binding inhibitors. The acidic region lying upstream of the hyalomin-1 cleavage site is somewhat conserved in peptides from other tick species (Fig 1), and interactions with this sequence may explain the salt dependence of hyalomin-1 binding. In the absence of the C-terminal sequence, however, this region of the peptide is not inhibitory and does not bind with thrombin at the relatively high concentrations tested here. It is not known if tyrosine sulfation this acidic region could be modified *in vivo*, but testing of a sulfated variant of the 13–44 peptide suggests that this does not dramatically increase potency. Apparently,the N-terminal regions do not constitute an independent exosite-binding domain.

## Supporting Information

S1 ARRIVE Checklist The Arrive Guidelines Checklist for in vivo animal research. (PDF)
